# Preschool Gender-Typed Play Behavior Predicts Adolescent Gender-Typed Occupational Interests: A 10-Year Longitudinal Study

**DOI:** 10.1007/s10508-021-01976-z

**Published:** 2021-03-22

**Authors:** Karson T. F. Kung

**Affiliations:** 1 Department of Psychology, University of Hong Kong, Pokfulam, Hong Kong; 2 Department of Psychology, University of Cambridge, Cambridge, UK; 3 School of Psychology, University of Kent, Canterbury, UK

**Keywords:** Sex, Gender, Play, Occupation, Adolescence, ALSPAC

## Abstract

There are significant gender differences in both play behavior and occupational interests. Play has been regarded as an important medium for development of skills and personal characteristics. Play may also influence subsequent preferences through social and cognitive processes involved in gender development. The present study investigated the association between gender-typed play behavior in early childhood and gender-typed occupational interests in early adolescence. Participants were drawn from a British longitudinal population study, the Avon Longitudinal Study of Parents and Children. Participants were recruited based on their parent-reported gender-typed play behavior assessed at age 3.5 years. There were 66 masculine boys and 61 masculine girls, 82 feminine boys and 69 feminine girls, and 55 randomly selected control boys and 67 randomly selected control girls. At age 13 years, the participants were administered a questionnaire assessing their interest in gender-typed occupations. It was found that masculine children showed significantly more interest in male-typical occupations than did control or feminine children. Compared with control children, feminine children had marginally significantly lower interest in male-typical jobs. Masculine children also had significantly lower interest in female-typical jobs than did control or feminine children. The associations were not moderated by gender and were observed after taking into account sociodemographic background, parental occupations, and academic performance. The degree of gender-typed play shown by preschoolers can predict their occupational interests 10 years later following transition into adolescence. Childhood gender-typed play has occupational implications that transcend developmental stages.

## Introduction

Average gender differences in preferences for certain toys emerge by the end of the first year of life (Alexander et al., 2009; Jadva et al., 2010; Lauer et al., 2018). Girls prefer domestic toys and dolls, whereas boys prefer toy vehicles and weapons (Davis & Hines, 2020; O’Brien & Huston, 1985; Pasterski et al., 2005; Ruble et al., 2006). In addition, gender-segregated play is commonly observed among toddlers and older children (Maccoby & Jacklin, 1987). Boys and girls play more frequently with peers of their own gender than of the other gender (Maccoby, 1998). There are also average differences in boys’ and girls’ play styles. Girls engage in verbal and nurturant styles of play, while boys play in a more active and physical fashion (Maccoby, 1998; Pasterski et al., 2011).

Gender differences are also evident in occupations and occupational interests. Across the globe, women represented only 27% of all national parliamentarians in 2017 (Inter-Parliamentary Union, 2018). In the UK, only 23% of the Science, Technology, Engineering, and Mathematics (STEM) workforce were women in 2017 (WISE, 2017). By contrast, 72% of school teachers, 86% of nurses, and 92% of secretaries were women in the UK in 2017 (UK Office for National Statistics, 2017). Gender differences in occupations are reflected in individuals’ occupational interests. Gender-typed occupational interests have been found in preschoolers, school-aged children, adolescents, and adults (Hilliard & Liben, 2010; Lamb et al., 2009; Liben & Bigler, 2002; Weisgram et al., 2010). For example, starting from the preschool years, girls are more interested in becoming cheerleaders, nurses, florists, and hairstylists, whereas boys are more interested in becoming scientists, police officers, ship captains, and computer engineers (Hilliard & Liben, 2010; Lamb et al., 2009; Liben & Bigler, 2002; Weisgram et al., 2010).

Play has been proposed as an important daily medium for children to learn and practice knowledge and roles (Cherney & London, 2006; Miller, 1987; Weisgram & Dinella, 2018). Qualitative differences of male- and female-typical play may contribute to the development of differential personal characteristics and skills associated with male- and female-typical occupations. Male-typical occupations tend to be realistic, risky, and things-oriented and female-typical occupations tend to involve fashion, children, and helping others (Lippa, 1998, 2005). Similarly, male-typical play is perceived as more competitive, risky, and involving more construction and female-typical play is perceived as having a stronger focus on physical appearance, nurturance, and domestic skills (Blakemore & Centers, 2005). In addition, it has been argued that strong visuospatial skills are important for success in certain male-typical jobs such as those in the STEM fields (Shea et al., 2001; Sorby & Baartmans, 2000) and empathy and social skills have been associated with caring occupations that are female-typical (Mercer & Reynolds, 2002; Reynolds & Scott, 1999). Research has shown that these cognitive and social skills are malleable and can be improved by training (Brunero et al., 2010; Satterfield & Hughes, 2007; Uttal et al., 2013). It has been proposed that male-typical play may elicit more spatial activities and female-typical play may elicit more nurturing behavior (Cherney & London, 2006; Miller, 1987). It has also been found that children who engage in more female-typical play show better comforting skills (Li & Wong, 2016) and children who engage in more male-typical play show better visuospatial skills (Jirout & Newcombe, 2015).

In addition to personal characteristics and skills, play may influence subsequent occupational interests via cognitive and social mechanisms involved in gender development. Cognitive perspectives suggest that children’s decisions to approach or avoid a gender-related activity are dependent on their knowledge of what is male-typical and female-typical, as well as their self-identification as boys or girls (Martin & Halverson, 1981; Martin et al., 2002). One key aspect of gender identity is self-perceived gender typicality and individuals may base their self-perceived gender typicality on their own gender-typed behavior (Egan & Perry, 2001). Children who engage in behavior more typical of their own gender show higher levels of self-perceived gender typicality than those who engage in behavior that is less typical of their own gender (Egan & Perry, 2001). According to theories focusing on the role of gender schemata, such as the gender schema theory (Bem, 1981; Martin & Halverson, 1981), children with high levels of gender-typed behavior and self-perceived gender typicality may maintain gender-typed preferences through acquiring gender-related information and assimilating such information into one’s self-concept, whereas children who are less gender-typed may possess a more flexible gender schema system. Similarly, the dual-pathway model proposes that children may use their gender schemata as a filter to assess whether an event is gender salient (i.e., gender salience filter) before making a decision on whether they want to engage with the event (Liben & Bigler, 2002). Gender-typed play may provide a platform for children to acquire and reinforce gender schemata, which may affect their subsequent preferences.

Social and sociocognitive perspectives suggest that children can construct their own social environments by choice of playmates and that children model peers in their environments to shape subsequent gender development (Bussey & Bandura, 1999; Maccoby, 1998). Notably, children do not only select playmates based on peers’ gender, but also peers’ similarities to themselves in terms of levels of gender-typed activities (Martin et al., 2013). Peers might also enforce gender norms (Xiao et al., 2019). Taken together, it is possible that childhood gender-typed play behavior contributes to the development of gender-related identity, schema, environment, and socialization, which in turn shape subsequent gender-typed occupational interests. For example, boys and girls having relatively high levels of male-typical play behavior may perceive themselves as more male-typical, interact more often with male-typical peers, and seek male-typical experiences, resulting in some continuity of male-typical behavior and preferences across developmental stages.

The present study aimed to investigate whether preschool gender-typed play behavior predicts gender-typed occupational interests in adolescence. Contemporaneous research has shown that gender-typed play and occupational interests are related in preschool and school-aged children (Coyle & Liben, 2016; Lamb et al., 2009; Weisgram et al., 2010), but there is a lack of longitudinal research examining the association across developmental stages. In the present study, participants were drawn from the Avon Longitudinal Study of Parents and Children (ALSPAC). The ALSPAC is a longitudinal population study in England that employed the Pre-School Activities Inventory (PSAI; Golombok & Rust, 1993a, b) to measure gender-typed play behavior. Based on PSAI scores at age 3.5 years, subsamples of masculine boys and girls, feminine boys and girls, and randomly selected control boys and girls were recruited. At age 13 years, the selected individuals were asked to complete the occupations subscale of the Children’s Occupations, Activities, and Traits-Personal Measure (COAT-PM; Liben & Bigler, 2002). This subscale measures adolescents’ interest in male-typical and female-typical occupations. It was hypothesized that masculine children at age 3.5 years would show more interest in male-typical occupations and less interest in female-typical occupations at age 13 years in comparison with control children and feminine children. It was also hypothesized that feminine children at age 3.5 years would show less interest in male-typical occupations and more interest in female-typical occupations at age 13 years in comparison with control children and masculine children. Since prior contemporaneous research did not report any gender-specific effects on the relationship between gender-typed play and gender-typed occupational interests (e.g., Liben & Bigler, 2002), no interactions with gender were hypothesized.

## Method

### Participants and Procedure

The participants were drawn from the ALSPAC, a longitudinal population study in the UK, with over 14,000 mothers and their children enrolled prenatally (Boyd et al., 2013; Fraser et al., 2013). The ALSPAC enrolled pregnant women who lived within Avon, a geographically defined area in Southwest England, and had expected delivery dates between April 1, 1991, and December 31, 1992 (Boyd et al., 2013).

Due to practical constraints, it was not possible for the current study to follow up all the participants from the entire ALSPAC. Using gender-typed play behavior assessed at age 3.5 years, the current study selected and followed up a subsample of the children at age 13 years. Six groups of children were selected at age 3.5 years, including boys (*n* = 122) and girls (*n* = 111) with relatively masculine scores (masculine children), boys (*n* = 110) and girls (*n* = 109) with relatively feminine scores (feminine children), and randomly selected boys (*n* = 99) and girls (*n* = 108) (control children). In the current study, 66 masculine boys and 61 masculine girls, 82 feminine boys and 69 feminine girls, and 55 control boys and 67 control girls completed the occupational interests measure at age 13 years, representing 61% of the sample selected at age 3.5 years. The participation rate in the current study was slightly higher than that in the entire ASLPAC (54% of all adolescents continued to participate in other aspects of ALSPAC at age 13 years). In terms of withdrawal, there were no significant differences among groups of girls, or between control and masculine boys, but feminine boys were significantly more likely to participate at age 13 years than control or masculine boys. There was no significant difference between boys and girls in the distribution of membership across the masculine, feminine, and control groups. To be classified as masculine or feminine, the children had to have PSAI scores that were at least 1 *SD* from the standardized norms for their own gender. The control children were randomly selected from the rest of the study population. Mean PSAI scores for the control children were close to means of the standardized population norms for their own gender in the original PSAI study (Golombok & Rust, 1993a, b), suggesting that control boys’ and girls’ gender-typed play behavior was in line with the average of the general population. Within each gender, there was no overlap in the ranges of PSAI scores across the masculine, control, and feminine groups. Descriptive statistics of PSAI scores for all of the groups are shown in Table 1.

**Table 1:** Descriptive statistics of the Pre-School Activities Inventory (PSAI) scores and the Occupations subscale of the Children’s Occupations, Activities, and Traits-Personal Measure (COAT-PM) scores within each gender and each childhood gender-typed play behavior group

	Boys *n* = 203	Girls *n* = 197	Masculine boys *n* = 66	Control boys *n* = 55	Feminine boys *n* = 82	Masculine girls *n* = 61	Control girls *n* = 67	Feminine girls *n* = 69
	*M* (*SD)*	*M* (*SD)*	*M* (*SD)*	*M* (*SD)*	*M* (*SD)*	*M* (*SD)*	*M* (*SD)*	*M* (*SD)*
PSAI	60.59 (16.38)	35.59 (17.12)	80.70 (4.60)	61.21 (5.96)	43.98 (4.44)	56.80 (4.76)	35.31 (7.72)	17.12 (4.14)
Male-typical occupations	0.25 (0.27)	− 0.19 (0.23)	0.34(0.24)	0.26 (0.26)	0.18 (0.29)	− 0.15 (0.21)	− 0.20 (0.23)	− 0.21 (0.25)
Female-typical occupations	− 0.33 (0.21)	0.14 (0.26)	− 0.39 (0.22)	− 0.32 (0.22)	− 0.28 (0.20)	0.05 (0.23)	0.16 (0.25)	0.19 (0.27)

The standardized norm of the PSAI is *M* = 60, *SD* = 10 for boys and *M* = 40, *SD* = 10 for girls (Golombok & Rust, 1993a, b)

The current age 13 sample was representative of the geographic area of Avon and diverse in socioeconomic background. With regard to ethnicity, around 96% of the children were Caucasian. In terms of paternal occupation, around 50% of the sample was professional/managerial and around 50% was skilled/partly skilled/unskilled. In terms of maternal occupation, around 40% of the sample was professional/managerial and around 60% was skilled/partly/skilled/unskilled. Ethical approval for the study was obtained from the ALSPAC Ethics and Law Committee and the Local Research Ethics Committees. Please note that the study Web site contains details of all the data that are available through a searchable data dictionary (http://www.bris.ac.uk/alspac/).

### Measures

#### Preschool Gender-Typed Play Behavior

At age 3.5 years, parents completed the Pre-School Activities Inventory (PSAI; Golombok & Rust, 1993a, b). The measure contains a list of 24 items addressing levels of gender-typed play behavior exhibited by children (e.g., playing with dolls, guns, and swords; playing with girls; pretending to be a female character; enjoying rough-and-tumble play). Parents were asked to report on their children’s behavior on a five-point Likert scale. Each item has a score of 1 to 5, representing the response categories “never,” “hardly ever,” “sometimes,” “often,” and “very often.” There are 12 male-typical items and 12 female-typical items. Using the standard scoring formula, the female-typical items were reversed scored and pseudo *T*-scores were generated; higher PSAI scores indicate more male-typical and less female-typical play behavior, whereas lower PSAI scores indicate more female-typical and less male-typical play behavior (Golombok & Rust, 1993a):

$$ {\text{Score}}\, = \,48.25\, + \,1.1\, \times \,\left( {{\text{the\,sum\,of\,``male''\,items}} - {\text{sum\,of\,``female''\,items}}} \right). $$

The PSAI was psychometrically constructed and validated in children aged 2 to 6 years to measure gender-typed play as a single trait. In the standardization sample, internal consistency was .88 and test–retest reliability was .84, and factor analysis supported a one-factor model (Golombok & Rust, 1993a). In the current sample, internal consistency was high (.94) and confirmatory factor analysis with residual covariance allowed testing the one-factor model showed adequate model fit, *χ*^2^(207) = 543.47, *p* < .001, CFI = .94, RMSEA = .06 [.06, .07]. The PSAI contains an item assessing play behavior that involves a male-typical occupation. Given that scoring with and without the occupation item generated almost identical scores in the current sample (*r* = 0.99) and that analyses based on scoring without the occupation item did not change the results or conclusions of the current study, the standard scoring method based on all the items was used.

#### Adolescent Gender-Typed Occupational Interests

At age 13 years, participants were administered the occupations form of the Children’s Occupations, Activities, and Traits-Personal Measure Short Form (COAT-PM; Liben & Bigler, 2002). The measure contains a list of ten male-typical occupations (e.g., architect, banker, computer builder, scientist, ship captain) and ten female-typical occupations (e.g., florist, hair stylist, jewelry maker, nurse, secretary). Participants were asked how much they would want to do each job on a four-point Likert scale. Each item has a score of 1 to 4, representing the response categories “not at all,” “not much,” “some,” and “very much.” In accordance with prior research on gender-typed occupational interests (e.g., Dinella et al., 2014; Lippa, 2010), ipsatization was applied to correct for an elevated response set (tendency for individuals to show interest in many or few occupations); individuals’ mean scores of all items were subtracted from scores of each item. Summary scores for the two subscales were then computed by averaging ipsatized item scores. Higher scores indicate higher levels of interest. The measure was psychometrically constructed and validated in children aged 11–13 years. In the standardization sample, internal consistencies were .78 and .82 and test–retest reliabilities were .73 and .82 for the male-typical and female-typical occupations subscales, respectively (Liben & Bigler, 2002). In the current sample, internal consistencies were .73 for male-typical occupation items and .78 for female-typical occupation items.

#### Control Variables

Eight sociodemographic variables were considered: (1) maternal age at delivery; (2) maternal education; (3) family income; (4) mother’s occupation; (5) father’s occupation; (6) number of sisters; (7) number of brothers; and (8) academic performance. There were five levels for both maternal education and family income, with 5 being the highest (maternal education: university degree; family income: > £400 per week) and 1 being the lowest (maternal education: Certificate of Secondary Education (CSE); family income: £100 per week). There were four levels for occupations, with 4 being the highest (professional/managerial) and 1 being the lowest (partly skilled or unskilled). Academic performance was assessed on a five-point scale via both parent reports (5 = much above average; 1 = much below average) and child reports (5 = much better than other kids; 1 = much worse than other kids).

## Results

### Initial Analyses

None of the variables had any outliers (*z* =  ± 3.29), except that there were two outliers in the number of sisters. The two extreme values were winsorized. All variables were normally distributed (skewness =  ± 1).

Independent samples *t*-tests and Cohen’s *d* statistic were used to assess the significance and magnitude of gender differences in COAT-PM scores. Positive *d* values indicate stronger interest shown by boys, and negative values indicate stronger interest shown by girls. There were significant gender differences for male-typical occupations, *t*(398) = 17.52, *p* < .001, *d* = 1.75, and female-typical occupations, *t*(398) =  − 19.81, *p* < .001, *d* = − 1.98. Descriptive statistics of COAT-PM scores for different groups are shown in Table 1.

Associations between COAT-PM scores and control variables were examined using Pearson’s correlation analyses. These correlations are shown in Table 2. Control variables that had a significant correlation with COAT-PM scores were subsequently included in analyses of covariance (ANCOVAs) as covariates. For male-typical occupational interests, maternal education, maternal occupation, and self-reported academic performance were included as covariates. For female-typical occupational interests, maternal age at delivery, maternal education, maternal occupation, and self-reported academic performance were included as covariates. There were no significant correlations between occupational interests and other control variables, such as paternal occupation or the number of brothers or sisters, and thus, these other variables were not included as covariates in subsequent analyses.

**Table 2:** Correlations between control variables and scores on the Occupations subscale of the Children’s Occupations, Activities, and Traits-Personal Measure (COAT-PM)

	Male-typical occupations	Female-typical occupations
Maternal age at delivery (*n* = 400)	.05	− .10*
Maternal education (*n* = 391)	.11*	− .18***
Family income (*n* = 346)	− .01	.04
Maternal occupation (*n* = 373)	.11*	− .15**
Paternal occupation (*n* = 345)	.05	− .06
Number of sisters (*n* = 400)	.01	− .02
Number of brothers (*n* = 400)	.01	.01
Self-reported academic performance (*n* = 397)	.10*	− 11*
Parent-reported academic performance (*n* = 398)	− .04	− .02

*p < .05; **p < .01; ***p < .001

### Preschool Gender-Typed Play Behavior and Adolescent Occupational Interests

A series of 2 (Gender) × 3 (Preschool Gender-Typed Play Behavior) ANCOVAs were conducted to examine whether masculine, feminine, and control children, grouped based on their gender-typed play at age 3.5 years, differed in COAT-PM scores at age 13 years. Effect sizes of the main effects, interactions, and covariates were calculated using the *η*^2^ statistic, which indicates the percentage of variance explained. Simple contrasts were conducted to follow up any main effects of preschool gender-typed play behavior to determine whether there were differences between masculine and control children, between feminine and control children, and between masculine and feminine children. Magnitudes of these pairwise gender-typed play group differences were calculated using the Cohen’s *d* statistic.

#### Male-Typical Occupations

A significant effect of gender was found, *F*(1, 354) = 295.32, *p* < .001, *η*^2^ = .46, such that boys scored higher than girls. There was also a significant effect of preschool gender-typed play, *F*(2, 354) = 7.24, *p* < .01, *η*^2^ = .04. Simple contrasts showed that masculine children had significantly higher scores than control children, *p* < .05, *d* = 0.27, and feminine children, *p* < .001, *d* = 0.47. Control children had higher scores than feminine children, and this difference approached statistical significance, *p* < .10, *d* = 0.22. The interaction between preschool gender-typed play behavior and gender was not significant, *F*(1, 354) = 2.46, *p* = .10, *η*^2^ = .01. Two covariates were significant: maternal occupation: *F*(1, 354) = 4.26, *p* < .05, *η*^2^ = .01, and self-reported academic performance: *F*(1, 354) = 11.03, *p* < .01, *η*^2^ = .03, suggesting higher scores in children whose mothers had higher-status jobs and in children with higher self-reported academic performance. The third covariate was nonsignificant: maternal education: *F*(1, 354) = 0.23, *p* = .63, *η*^2^ < .01.

#### Female-Typical Occupations

A significant effect of gender was found, *F*(1, 353) = 363.74, *p* < .001, *η*^2^ = 0.51, with girls scoring higher than boys. There was also a significant effect of preschool gender-typed play, *F*(2, 353) = 11.82, *p* < .001, *η*^2^ = .06. Simple contrasts showed that masculine children had significantly lower scores than control children, *p* < .01, *d* = 0.45, and feminine children, *p* < .001, *d* = 0.62. Control children had lower scores than feminine children, although the difference was not statistically significant, *p* = .20, *d* = 0.16. The interaction between preschool gender-typed play behavior and gender was not significant, *F*(2, 353) = 0.11, *p* = .90, *η*^2^ < .01. Most of the covariates were significant: maternal education, *F*(1, 353) = 3.96, *p* < .05, *η*^2^ = .01; maternal occupation, *F*(1, 353) = 5.47, *p* < .05, *η*^2^ = .02; and self-reported academic performance: *F*(1, 353) = 11.51, *p* < .01, *η*^2^ = .03, suggesting higher scores in children whose mothers had lower levels of education qualifications and lower-status jobs and in children with lower self-reported academic performance. Maternal age at delivery was not significant, *F*(1, 353) = 0.33, *p* = .57, *η*^2^ < .01.

## Discussion

The present study found that preschool gender-typed play at age 3.5 years predicted subsequent gender-typed occupational interests in adolescence at age 13 years. There were significant gender differences in interest in male-typical and female-typical occupations in the expected directions. As hypothesized, there were significant group differences in interest in male-typical and female-typical occupations among children grouped as masculine, feminine, and control according to their preschool gender-typed play. Specifically, masculine children showed significantly more interest in male-typical occupations than did control or feminine children. Masculine children also showed significantly less interest in female-typical occupations than did control or feminine children. Compared with control children, feminine children had marginally significantly lower interest in male-typical occupations. Feminine children had more interest in female-typical occupations than did control children, but the difference was not statistically significant. Although not all the pairwise group comparisons based on preschool gender-typed play reached the conventional level of statistical significance, the trend for both male-typical and female-typical occupational interests across the groups was in line with the prediction. In addition, by Cohen’s criteria, some of the group differences were of a medium magnitude and the smallest group difference approached the small effect range. Furthermore, these 10-year longitudinal associations were observed after taking into account a range of sociodemographic variables, such as maternal age at delivery, parental education and occupation, and academic performance. While preschool gender-typed play did not explain as much variance in occupational interests as did gender, play accounted for more variance than did any of the sociodemographic control variables. There was no interaction between preschool gender-typed play and gender in predicting interest in male-typical or female-typical occupations, suggesting that the associations did not differ for boys and girls.

The current findings appear to support prior research, suggesting that gender-typed play may facilitate the development of personal characteristics and skills associated with male- and female-typical occupations (Blakemore & Centers, 2005; Cherney & London, 2006; Li & Wong, 2016; Lippa, 2005; Uttal et al., 2013). Findings from the present study also appear to concur with social, cognitive, and sociocognitive perspectives, suggesting that childhood gender-typed play may contribute to self-identification and self-socialization mechanisms involved in gender development, creating certain levels of continuity and stability in subsequent gender-typed preferences (Bussey & Bandura, 1999; Egan & Perry, 2001; Maccoby, 1998). These findings may also be interpreted to support theories focusing on gender schemata and the dual-pathway model (Bem, 1981; Liben & Bigler, 2002; Martin & Halverson, 1981). By showing that preschool gender-typed play predicts gender-typed occupational interests 10 years later in early adolescence, the current findings extend prior research reporting a contemporaneous link during childhood (Coyle & Liben, 2016; Liben & Bigler, 2002; Weisgram et al., 2010). Further research may measure and test the relative importance of different mechanisms via which childhood gender-typed play may contribute to subsequent gender-typed occupational interests.

It is noteworthy that adolescent occupational interests can have long-term implications for occupational choice. A meta-analysis found that occupational interests are consistent from early adolescence to middle adulthood (Low et al., 2005). Moreover, young people’s occupational interests can predict occupations up to 30 years later (Hansen, 2005; Rottinghaus et al., 2007). Further research may usefully examine the relationships among childhood play, adolescent occupational interests, and occupations in adulthood.

Further research may also explore how attempts to encourage boys and girls to engage more in gender-atypical play may affect subsequent occupational interests and choice. It has been suggested that reducing gender labels and gender-typed color coding may increase boys’ and girls’ interest in a wider range of toys, activities, and peer interactions (Hilliard & Liben, 2010; Weisgram et al., 2014; Wong & Hines, 2015; Yeung & Wong, 2018). Considering the importance of childhood play behavior in developing personal characteristics, skills, self-perception, and self-socialization, these strategies may reduce gender differences in occupation-related outcomes by creating more diverse learning opportunities for boys and girls and by helping them develop more flexible conceptions about gender typicality.

A limitation of the current longitudinal study is that preschool occupational interests were not controlled for. The ALSPAC study began in the early 1990s, and it appears that psychometrically validated and standardized measures of preschool gender-typed occupational interests were not available at that time. Further research is needed to empirically evaluate the relative contributions of play behavior and occupational interests in early childhood to subsequent occupational interests and choice. Nonetheless, it is unlikely that adolescent occupational interests are merely a product of preschool occupational interests. Theoretically, it has been proposed that characteristics and sociocognitive mechanisms associated with gender-typed play can contribute to subsequent gender-typed development and occupational interests. In addition, while the relationship between gender-typed play and occupational interests is potentially reciprocal in later stages of development, the relationship is more likely to be driven by play in the first place (i.e., play shaping occupational interests) than by occupational interests shaping play. It is firstly because some gender-typed play preferences emerge by a child’s first birthday, preceding the emergence of gender differences in occupational interests, and secondly because young children spend much of their daily life playing, but it is unlikely that they think about occupations frequently. The second argument also suggests that the potentially reciprocal relationship in later stages of development may be more strongly driven by play than occupational interests. Furthermore, although the two constructs may correlate with each other in different age groups, such contemporaneous and cross-sectional correlations do not guarantee cross-lagged longitudinal predictions. The current research contributes to the literature by showing that preschool gender-typed play can predict subsequent gender-typed occupational interests longitudinally across developmental stages.

Another limitation is that the current study employed a group comparison approach. Such an approach may limit the generalizability of the findings. It would be useful for further research using other designs and recruitment methods to try to replicate the longitudinal association between preschool gender-typed play and adolescent gender-typed occupational interests.

To conclude, using age-appropriate and psychometrically validated measures of gender-typed play and occupational interests, a relatively large study population, a l0-year longitudinal design, and a sample that was representative of the study location, the present study found a link between preschool gender-typed play and adolescent gender-typed occupational interests in boys and girls. These findings suggest that play may shape subsequent occupational interests via the development of skills and personal characteristics, as well as sociocognitive processes such as self-identification and self-socialization. This study suggests that childhood gender-typed play has occupational implications that transcend developmental stages.
